# Analysis of the mediating effect of invasive ruminative contemplation on the relationship between social support and non-suicidal self-injury behavior in depressed patients

**DOI:** 10.3389/fpsyt.2025.1733769

**Published:** 2026-01-15

**Authors:** Mei Tang, Li Tao, Jie Liu, Ni Tao, Hong Peng, Jing Gu

**Affiliations:** Department of Psychosomatic Medicine, Deyang People’s Hospital, Deyang, Sichuan, China

**Keywords:** depressed patients, intrusive rumination, mediating effect, non-suicidal self-injurious behavior, social support

## Abstract

**Objective:**

To explore the mediating mechanism of intrusive rumination between social support and non-suicidal self-injury (NSSI) behavior in patients with depression.

**Methods:**

Patients with depression admitted to our hospital from September 2023 to February 2024 were selected as the study subjects. A questionnaire survey was conducted using the General Information Questionnaire, Intrusive Rumination Scale, Perceived Social Support Scale, and Ottawa Self-Injury Inventory (OSI). Pearson correlation analysis and structural equation modeling (SEM) were used to test the mediating effect.

**Results:**

The scores for intrusive rumination, total social support, and NSSI behavior in 120 depressed patients were (15.71 ± 2.13), (47.85 ± 4.69), and (16.35 ± 2.65), respectively. NSSI behavior was negatively correlated with the total social support score and its three dimensions (*P*< 0.05), and positively correlated with the total intrusive rumination score (*P*< 0.05). Intrusive rumination showed a mediating effect of 27.40% between social support and NSSI behavior.

**Conclusion:**

Social support can influence NSSI behavior in depressed patients by regulating intrusive rumination. It is crucial to emphasize the assessment of intrusive rumination in clinical practice to reduce the occurrence of NSSI behavior.

## 1 Introduction

Depression is a common mental health disorder characterized by core symptoms such as persistent low mood, diminished interest or pleasure, self-negation, sleep disturbances, appetite changes, and impaired cognitive function. These symptoms severely impact an individual’s social functioning and quality of life (1). Globally, the prevalence of depression continues to rise, with the World Health Organization identifying it as a leading cause of disability. In China, alongside increasing social competition pressure and the accelerated pace of life, the incidence of depression has been climbing annually. It is particularly prevalent among adolescents and young adults, making it a significant public health concern (2).

Intrusive rumination, a subtype of rumination measured by tools like the Event-Related Rumination Inventory (ERRI), refers to the involuntary, repetitive, and intrusive recurrence of thoughts about negative events or emotions. Individuals find it difficult to disengage from these thoughts, which are often accompanied by intense anxiety, self-blame, and feelings of helplessness (3). Research indicates that intrusive rumination is prevalent among depressed patients. It not only exacerbates depressive symptoms but is also closely linked to reduced self-regulatory capacity and maladaptive coping strategies (4). In recent years, some scholars have further suggested that intrusive rumination may contribute to the onset of non-suicidal self-injury (NSSI) by intensifying emotional dysregulation and self-criticism (5).

Non-suicidal self-injury (NSSI) refers to the deliberate infliction of harm to one’s own body tissue in the absence of suicidal intent. Common forms include cutting, burning, hitting, and others (6). NSSI is particularly prevalent among adolescents and young adults with depression, often serving as a means of emotion regulation. In the long term, it may evolve into an addictive behavior and significantly increase the risk of future suicide (7). Research has found that NSSI is not only associated with an individual’s emotion regulation strategies but is also influenced by psychosocial factors (8). As a crucial psychosocial resource, social support encompasses both practical assistance and emotional understanding from family, friends, and other social relationships. It is widely recognized as a key factor in alleviating psychological stress and enhancing psychological resilience. Studies have confirmed (9) that depressed patients with higher levels of social support exhibit a significantly lower incidence of NSSI behaviors, suggesting that social support may play a protective role between depression and self-injurious behaviors (10).

Furthermore, social support has been found to be negatively correlated with rumination; high levels of social support help individuals disengage from negative thoughts and reduce intrusive rumination (11). Although existing studies have separately explored the pairwise relationships between social support, intrusive rumination, and NSSI, systematic research on the underlying mechanism involving all three remains insufficient. Therefore, this study aims to construct a structural equation model (SEM) to thoroughly investigate the intrinsic relationships and the mediating effect of Event-Related Rumination Inventory (ERRI) between social support and NSSI behaviors in depressed patients. The goal is to improve the mental health outcomes of depressed patients and reduce the occurrence of NSSI behaviors.

## 2 Materials and methods

### 2.1 Participants

This study is a cross-sectional survey. Inclusion Criteria: (1) Age ≥ 18 years; (2) All patients met the diagnostic criteria for depression (12); (3) Hamilton Depression Scale score ≤ 20 points (13); (4) Patients in a stable recovery phase after treatment; (5) Ability to communicate normally and independently complete the scales; (6) Normal physical function; (7) Patients provided informed consent for the study. Exclusion criteria: (1) Patients with malignant tumors; (2) Patients with dependencies on drugs, alcohol, etc.; (3) Patients with poor compliance; (4) Patients with incomplete data. This study was approved by the Ethics Committee of our hospital (Approval No: 2025-04-070-K01), and all participating patients signed informed consent forms.

### 2.2 Sample size

Calculation The sample size for this study was estimated based on the principle in multivariate statistical analysis that the sample size should be 5–10 times the number of independent variables (14). The study involved variables including general information, social support, intrusive rumination, and NSSI behavior, totaling 21 independent variables. The minimum sample size was calculated as 21 × 5 = 105 cases. Considering the possibility of invalid questionnaires during the collection process, the sample size was expanded to 120 cases, meeting the minimum requirement for sample size estimation and satisfying the needs of structural equation modeling analysis.

### 2.3 Study tool

#### 2.3.1 General information questionnaire

A self-designed questionnaire by the researchers, covering sociodemographic characteristics such as gender, age, and place of residence.

#### 2.3.2 Intrusive rumination

Assessed using the Chinese version of the Event-Related Rumination Inventory (ERRI) translated by Jia Dengshuai et al. (15). The inventory consists of two subscales: intrusive rumination and deliberate rumination, with a total of 20 items rated on a 4-point Likert scale. This study focused on the intrusive rumination subscale, which includes 10 items and has a total score range of 0–30. A higher score indicates a higher level of individual cognitive processing. In this study, the Cronbach’s α coefficient for the intrusive rumination subscale was 0.921.

#### 2.3.3 Perceived social support

Measured using the Chinese version of the Perceived Social Support Scale (PSSS) translated by Fang Tingting (16). The scale comprises three dimensions: family support, friend support, and other support, with a total of 12 items rated on a 7-point scale. The total score ranges from 12 to 84, with scores below 50 indicating low social support, scores between 50 and 68 indicating moderate social support, and scores above 68 indicating high social support. Higher scores reflect a higher level of perceived social support. In this study, the Cronbach’s α coefficient for the PSSS was 0.901.

#### 2.3.4 Non-suicidal self-injury

Evaluated using the Ottawa Self-Injury Inventory (OSI) (17), which was adapted to the participants’ specific circumstances. The scale assesses the frequency of NSSI behaviors over the past 12 months and includes 10 types of self-injurious behaviors (e.g., pulling one’s hair forcefully, scratching oneself, banging one’s head against a wall). The frequency of each behavior is scored as follows: 0 times (0 points), 1 time (1 point), 2–4 times (2 points), and ≥5 times (3 points). According to the NSSI criteria (18), a total score greater than 0 indicates the presence of NSSI behavior. The severity of NSSI is further classified as mild (1–10 points), moderate (11–20 points), or severe (21–30 points). In this study, the Cronbach’s α coefficient for the OSI was 0.853.

### 2.4 Data collection

As a cross-sectional study, data were collected through face-to-face questionnaire surveys administered to depressed patients during their treatment recovery phase. All investigators received unified standardized training to thoroughly master the questionnaire content. Patients meeting the inclusion criteria were selected to complete the questionnaires. When patients had questions regarding the questionnaire, detailed explanations were provided without influencing their response choices. A total of 130 questionnaires were distributed, with 120 valid questionnaires recovered, resulting in an effective response rate of 92.31%.

### 2.5 Statistical analysis

SPSS 25.0 software was employed for statistical analysis. Normally distributed categorical data were described using frequencies and percentages, while continuous data were presented as mean ± standard deviation ( $\bar{X}$ ± *s*). Pearson correlation analysis was used to examine the relationships between ERRI, social support, and NSSI behaviors in depressed patients, with a significance level set at α=0.05. Amos 21.0 software was utilized to construct a structural equation model (SEM) and test for mediating effects. The non-parametric percentile Bootstrap method was applied for mediation effect testing with 5000 repeated samplings. Effect sizes and their 95% confidence intervals (CI) were calculated. If the 95% CI of the direct and indirect effects did not include zero, the mediating effect was considered statistically significant. A two-tailed *P*< 0.05 was considered statistically significant.

## 3 Results

### 3.1 General clinical data of the included patients

A total of 120 patients with depression were included in this study. Detailed demographic and clinical characteristics are summarized in **Table 1**.

**Table 1:** Clinical and demographic characteristics.

Item	Category	n (%)
Gender	Male	52 (43.33)
	Female	68 (56.67)
Age (years)	≥ 25	62 (51.67)
	< 25	58 (48.33)
Family Conflict	Yes	73 (60.83)
	No	47 (39.17)
Place of Residence	Urban	99 (82.50)
	Rural	21 (17.50)
Academic Pressure	Yes	59 (49.17)
	No	61 (50.83)
Employment Pressure	Yes	80 (66.67)
	No	40 (33.33)
Religious Belief	Yes	35 (29.17)
	No	85 (70.83)
Family Economic Status	Poor	69 (57.50)
	Average	35 (29.17)
	Good	16 (13.33)

### 3.2 Scores of intrusive rumination, social support, and non-suicidal self-injury in depressed patients

Among the 120 depressed patients, the ERRI score was 15.71 ± 2.13, the total social support score was 47.85 ± 4.69, and the NSSI behavior score was 16.35 ± 2.65. See **Table 2** for details.

**Table 2:** Scores for intrusive rumination, social support, and non-suicidal self-injury behavior in depressed patients (points).

Item	Total score range	Score (χ̄ ± s)	Average item score (χ̄ ± s)
ERRI	0-30	15.71 ± 2.13	1.57 ± 0.53
Total PSSS Score	12-84	47.85 ± 4.69	3.99 ± 1.28
Family Support	4-28	16.34 ± 2.04	4.09 ± 1.31
Friend Support	4-28	17.24 ± 2.12	4.31 ± 1.23
Other Support	4-28	16.96 ± 2.04	4.24 ± 1.51
NSSI Behavior	0-30	16.35 ± 2.65	1.64 ± 0.55

### 3.3 Correlation analysis of non-suicidal self-injury with social support and intrusive rumination in depressed patients

NSSI behavior was significantly correlated with both social support and ERRI (*P*< 0.05). Specifically, NSSI behavior was negatively correlated with the total social support score and its three dimensions (*P*< 0.05), and positively correlated with the total ERRI score (*P*< 0.05). See **Table 3** for details.

**Table 3:** Correlation analysis of non-suicidal self-injury behavior with social support and intrusive rumination in depressed patients (r values).

Variable	Intrusive rumination	Total PSSS score	Family support	Friend support	Other support	NSSI behavior
Intrusive Rumination	1					
Total PSSS Score	-0.621**	1				
Family Support	-0.597**	0.593**	1			
Friend Support	-0.564**	0.579**	0.612**	1		
Other Support	-0.586**	0.567**	0.441**	0.512**	1	
NSSI Behavior	0.731**	-0.697**	-0.612*	-0.648**	-0.671**	1

*P< 0.05, **P< 0.01.

### 3.4 Mediating effect of intrusive rumination between social support and non-suicidal self-injury in depressed patients

**Figure 1:**
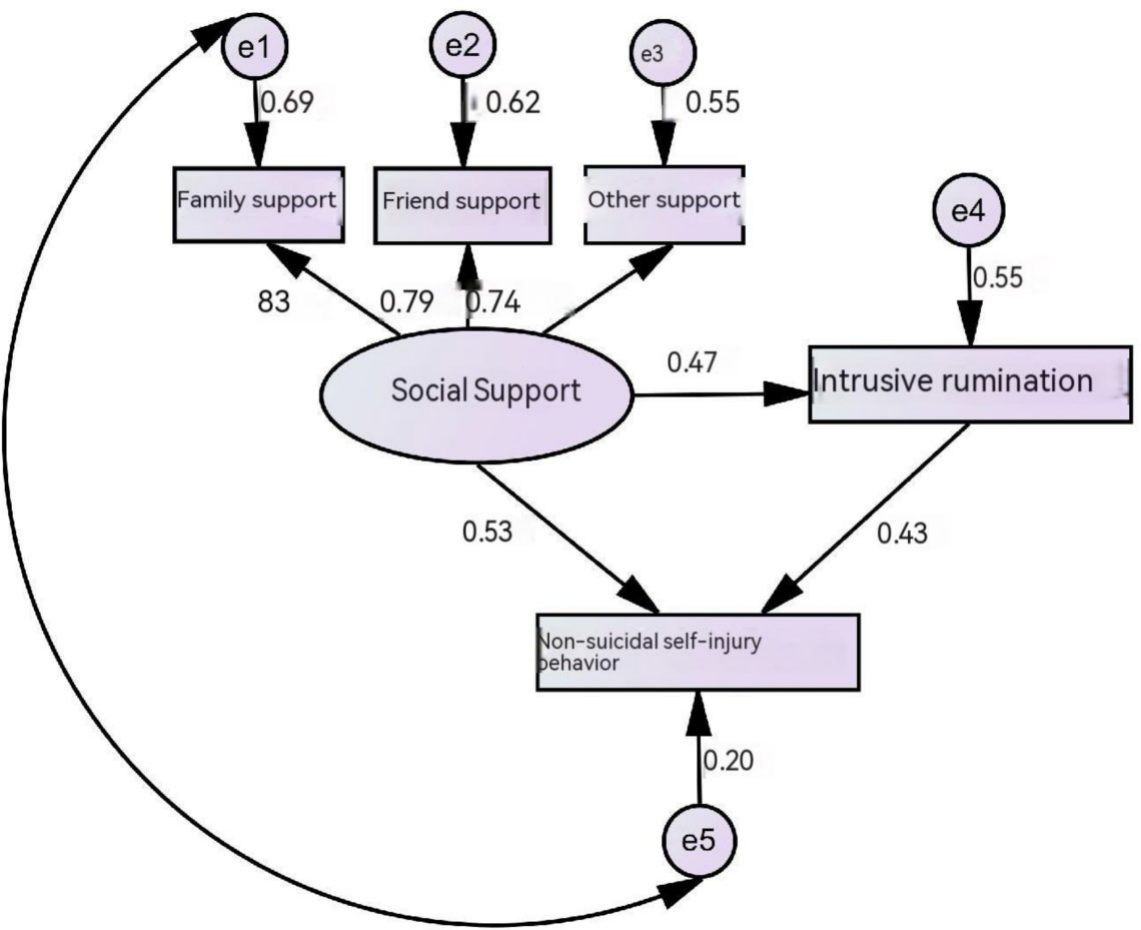
Mediating effect model of social support and intrusive rumination on non-suicidal self-injury in depressed patients.

A structural equation model (SEM) was constructed to examine the pathway relationships among ERRI, social support, and NSSI behaviors, with a specific focus on the mediating role of ERRI between social support and NSSI behaviors. In the model, NSSI behavior was set as the dependent variable, social support as the independent variable, and ERRI as the mediating variable, with the dimensions of each scale serving as observed variables. The model parameters were estimated using the maximum likelihood (ML) method. After model adjustment, the fit indices were as follows: χ² = 48.853, *P* > 0.05 (χ²/df = 1.388), indicating a well-fitting model. The model is shown in **Figure 1**.

### 3.5 Effect relationships

The mediating effects were decomposed into direct and indirect effects. The direct effects indicated that social support had a positive direct effect on NSSI behavior (effect size = 0.53), a positive direct effect on ERRI (effect size = 0.47), and ERRI had a positive direct effect on NSSI behavior (effect size = 0.43). The indirect effect revealed that social support also influenced NSSI behavior indirectly through ERRI, with an indirect effect size of 0.47 × 0.43 = 0.20. The proportion of the total effect mediated by ERRI was calculated as 0.20/(0.20 + 0.53) = 27.40%, indicating a partial mediating effect of ERRI in the relationship between social support and NSSI behavior. See **Table 4** for path coefficients.

**Table 4:** Path coefficients for non-suicidal self-injury behavior, social support, and intrusive rumination in depressed patients.

Influence path	Effect value	Mediating effect percentage	P value
Social Support → Non-Suicidal Self-Injury Behavior	0.53	–	<0.05
Social Support → Intrusive Rumination	0.47	–	<0.05
Intrusive Rumination → Non-Suicidal Self-Injury Behavior	0.43	–	<0.05
Social Support → Intrusive Rumination → Non-Suicidal Self-Injury Behavior	0.20	27.40%	

## 4 Discussion

### 4.1 Non-suicidal self-injury behavior in depressed patients at a moderate level

Non-suicidal self-injury (NSSI) is a common and complex psychological issue among depressed patients, representing a frequent form of self-harm behavior in this population. Studies have indicated (19) that 44% of depressed patients exhibit NSSI behaviors, which not only inflict physical and psychological harm on the individuals themselves but also impose negative impacts on their families and society.

The results of this study show that the NSSI behavior score among the 120 depressed patients was 16.35 ± 2.65, indicating a moderate level. This highlights the need for increased attention to NSSI behaviors in depressed patients and calls for enhanced research and clinical practices to improve understanding and management of this issue. Some studies have suggested (20) that self-injurious behavior may be regarded as an addictive behavior, as individuals gradually develop increased tolerance to pain through self-harm, leading to reduced fear of death and consequently elevating the risk of suicide.

The prevalence of such behaviors may be attributed to factors such as rising social pressures, accelerated pace of life, and interpersonal tensions, which drive an increasing number of depressed patients to resort to NSSI as a means of releasing inner suffering and anxiety. Many young individuals, when confronted with academic pressure, family conflicts, or emotional distress, turn to self-injury to alleviate psychological pain—a behavior particularly common among adolescents and young adults (21).

Moreover, depressed patients often experience comorbid psychological issues such as anxiety disorders and post-traumatic stress disorder. These conditions interact and exacerbate one another, increasing the patients’ vulnerability to self-injurious behaviors. Such NSSI behaviors not only intensify the psychological distress of depressed patients but also adversely affect treatment outcomes and recovery processes. Therefore, timely identification and intervention for NSSI behaviors in this population are of critical importance.

### 4.2 Correlation analysis of non-suicidal self-injury with social support and intrusive rumination in depressed patients

The results of this study indicate that NSSI behavior in depressed patients is significantly correlated with both social support and intrusive rumination (ERRI) (*P*< 0.05). Specifically, NSSI behavior showed a significant negative correlation with the total social support score and its three dimensions (*P*< 0.05), suggesting that higher levels of social support—including family, friend, and other support—are associated with lower NSSI behavior.

This finding is consistent with the research of Lu Jiahui et al. (22). Family support can provide emotional sustenance and understanding, making patients feel valued and cared for, thereby alleviating loneliness and self-blame and reducing self-injury tendencies. Friend support offers social companionship and assistance, helping patients build positive social connections, mitigate feelings of isolation and stress, and promote emotional release and regulation. Other forms of support, such as psychological counseling and professional treatment, can further provide cognitive and emotional guidance, enabling patients to better understand and cope with depression, thus decreasing the incidence of self-injury.

Simultaneously, a significant positive correlation was observed between NSSI behavior and the total ERRI score (*P*< 0.05), indicating that higher levels of intrusive rumination are associated with increased NSSI behavior. This aligns with the findings of Zhang Wenqing et al. (23). A tendency toward frequent negative self-reflection and persistent adverse thinking may lead to the accumulation of negative emotions and difficulties in emotion regulation, thereby elevating the risk of self-injury. Underlying psychological factors may include self-denial, despair about the future, and disillusionment with life.

Depressed patients often lack self-affirmation and confidence, excessively focusing on their own shortcomings and errors, which exacerbates negative emotions and self-doubt (24). Moreover, feelings of hopelessness and disappointment may leave individuals feeling helpless and trapped, making it challenging to adopt positive coping strategies and leading them to resort to self-injury as a means of emotional regulation (25). Therefore, interventions and psychotherapies targeting intrusive rumination are of great significance in reducing NSSI behavior among depressed patients.

### 4.3 The mediating role of intrusive rumination and its potential neurobiological mechanisms

This study confirms the mediating role of intrusive rumination between social support and NSSI behaviors, a psychological pathway that may have underlying neurobiological foundations.

In recent years, research in affective neuroscience has begun to link repetitive negative thinking, such as rumination, with the function of the brain’s glymphatic system. The glymphatic system is a waste-clearance mechanism in the brain, primarily active during sleep, responsible for removing neurotoxic substances, including β-amyloid and tau proteins. One theory suggests that impaired glymphatic function may exacerbate neuroinflammation and disrupt the function of brain regions involved in emotion regulation, such as the prefrontal cortex and anterior cingulate cortex, thereby increasing cognitive rigidity and the persistence of intrusive thoughts (26). A recent review further connects glymphatic dysfunction to rumination and emotional dysregulation in various psychiatric disorders, including depression, offering a novel biopsychosocial perspective on the understanding of ruminative thinking (27).

Additionally, research models propose that the efficiency of glymphatic clearance may influence the persistence of depression by modulating activity in neural circuits associated with rumination, such as the default mode network (28). This suggests that the buffering effect of social support on ruminative thinking observed in this study may be partially mediated by factors such as improved sleep quality, which optimizes brain clearance function and alleviates cognitive fixation. Therefore, future research integrating neuroimaging and fluid biomarkers could help provide a more comprehensive, multimodal understanding of the mechanisms through which psychosocial factors influence behavioral phenotypes via neurobiological pathways.

### 4.4 The mediating effect of social support and intrusive rumination on non-suicidal self-injury in depressed patients and corresponding nursing strategies

Using structural equation modeling and Bootstrap testing, this study confirmed that intrusive rumination plays a partial mediating role between social support and NSSI behavior, with an effect proportion of 27.40%.

This finding holds significant theoretical and clinical implications. Theoretically, it clearly reveals a potential psychopathological pathway: “insufficient social support → exacerbation of intrusive rumination → increased risk of NSSI behavior”. This aligns with the conceptual model proposed by Mendez et al. (29), which suggests that “social adversity leads to self-harm through cognitive-affective processing”.

However, by quantifying the mediating effect, this study provides empirical support for this theory within the population of depressed patients. At the clinical practice level, this mediating effect suggests that intervention efforts should shift from a single-dimensional approach to a multi-dimensional integrated strategy. First, directly targeting NSSI behavior, safety assessments and management protocols should be implemented, helping patients identify behavioral triggers and establish alternative emotion regulation techniques (e.g., vigorous exercise, snapping a rubber band on the wrist). Second, addressing the mediating mechanism—intrusive rumination—interventions such as metacognitive therapy (MCT) can be introduced to change patients’ relationship with ruminative thoughts, or mindfulness-based cognitive therapy (MBCT) can be adopted to cultivate awareness and a non-judgmental attitude toward negative thoughts, thereby breaking the automatic chain of rumination. Finally, targeting the root factor—social support—interventions should go beyond simply “encouraging socialization”.

Instead, family members should be guided to engage in effective, non-critical communication, and patients should be assisted in building genuine social connections to enhance their psychological resilience at the source. Future research could build on this foundation to design and validate integrated programs that combine social support enhancement and rumination intervention. Additionally, technologies such as neuroimaging could be utilized to explore how these interventions reshape the psychological-behavioral pathways revealed in this study, particularly from the perspective of brain functional connectivity.

## 5 Conclusion

In summary, this study found that the scores for intrusive rumination (ERRI), total social support, and non-suicidal self-injury (NSSI) behavior among depressed patients were all at moderate levels. NSSI behavior was negatively correlated with the total social support score and its three dimensions, while it was positively correlated with the total ERRI score. Importantly, ERRI demonstrated a partial mediating effect of 27.40% between social support and NSSI behavior, confirming that social support can influence NSSI behavior by regulating the tendency toward intrusive rumination. These findings suggest that clinical healthcare professionals should prioritize the assessment of intrusive rumination in depressed patients to mitigate the impact of insufficient social support on NSSI behavior.

## 6 Limitations

However, this study may be subject to sample selection bias, which could limit the generalizability of the results. Additionally, the data collection methods may introduce subjectivity and recall bias, potentially affecting the accuracy of the findings. Therefore, caution is advised when applying these results in clinical practice, and comprehensive analysis and decision-making tailored to individual circumstances are recommended.

## Data Availability

The raw data supporting the conclusions of this article will be made available by the authors, without undue reservation.
